# Transposition of the gastroduodenal artery for vascular reconstruction of invasive lesion of the hepatic hilum due to colorectal adenocarcinoma metastasis: case report

**DOI:** 10.1590/1677-5449.202400122

**Published:** 2025-01-13

**Authors:** Lucas Lourenço de Oliveira, Iasmin Maria Rodrigues Saldanha, Yago Eloy Souza Barbosa, Renato Mazon Lima Verde Leal, Abner Moreira Sampaio, Annya Costa Araújo de Macedo Goes, Marcelo Leite Vieira Costa

**Affiliations:** 1 Universidade Federal do Ceará – UFC, Hospital Universitário Walter Cantídio – HUWC, Fortaleza, CE, Brasil.; 2 Universidade Federal do Ceará – UFC, Fortaleza, CE, Brasil.

**Keywords:** hepatectomy, colorectal neoplasms, metastasectomy, vascular surgical procedures

## Abstract

Colorectal cancer is one of the most prevalent malignant neoplasms in Brazil. Hepatectomy for resection of liver metastases plays an essential role in increasing disease-free survival, with the possibility of cure. The feasibility of liver resection depends on factors related to the remaining liver after surgery. In this article, a case is reported on the hepatic metastasectomy approach in a 47-year-old female patient, with colon adenocarcinoma who underwent vascular reconstruction in the same surgical procedure. The intrahepatic mass was transposition of the gastroduodenal artery through the anastomosis of the gastroduodenal artery and the right hepatic artery in a continuous suture, without complications. It is concluded that vascular anastomosis in hepatectomies for colon adenocarcinoma metastases is a complex procedure that requires skill and experience from the surgeon. The success rate is high, but it is important to be aware of the risk factors for complications. The most current data suggest that vascular reconstruction does not alter overall disease-free survival, but further studies are needed.

## INTRODUCTION

Colorectal cancer (CRC) is the third most prevalent type of malignant neoplasm in Brazil (not including non-melanoma skin tumors). It is estimated that around 45,630 new cases will be diagnosed from 2023 to 2025, equating to an incidence of 21.1 cases per 100 thousand inhabitants.^1^ The most common site of metastasis is the liver, and data show that around half of CRC patients will have hepatic metastases during their clinical course.^2^

Hepatectomy for resection of liver metastasis play an essential role in extending disease-free survival, with the proven possibility of cure.^3^ Long-term survival after surgery for hepatic metastases of CRC has improved significantly. Over recent decades, as surgical techniques have improved, overall 5-year survival gains have reached 64% at some centers.^4^

The feasibility of hepatic resection depends on factors related to the liver remnant that will be left after surgery: preservation of vascular inflow and outflow; biliary drainage; and the possibility of preserving an adequate liver remnant, which is generally defined as a minimum of 20% for healthy liver or 30% after chemotherapy.^5^

The objective of this study is to report the management of a case of a female patient who underwent vascular transposition by anastomosis of the gastroduodenal artery and right hepatic artery during the same surgical procedure as a hepatectomy, because of tumoral involvement of a segment of the true hepatic artery.

This article was approved by the Research Ethics Committee under consolidated opinion n° 6.619.185 and Ethics Appraisal Submission Certificate 76809324.0.0000.5045.

## CASE REPORT

The patient was a 47-year-old female who sought medical attention for postprandial discomfort, dyspnea, asthenia, and increased abdominal volume with onset 4 months previously. She had a prior history of a moderately differentiated (pT2N0) adenocarcinoma of the colon that had been treated with proctosigmoidectomy in 2016, but she had not maintained clinical follow-up. She returned to follow-up in 2022, when computed tomography images were suggestive of metastasis restricted to the liver, located in the left lobe and measuring 15.7 cm × 10.5 cm.

**Figure 1:**
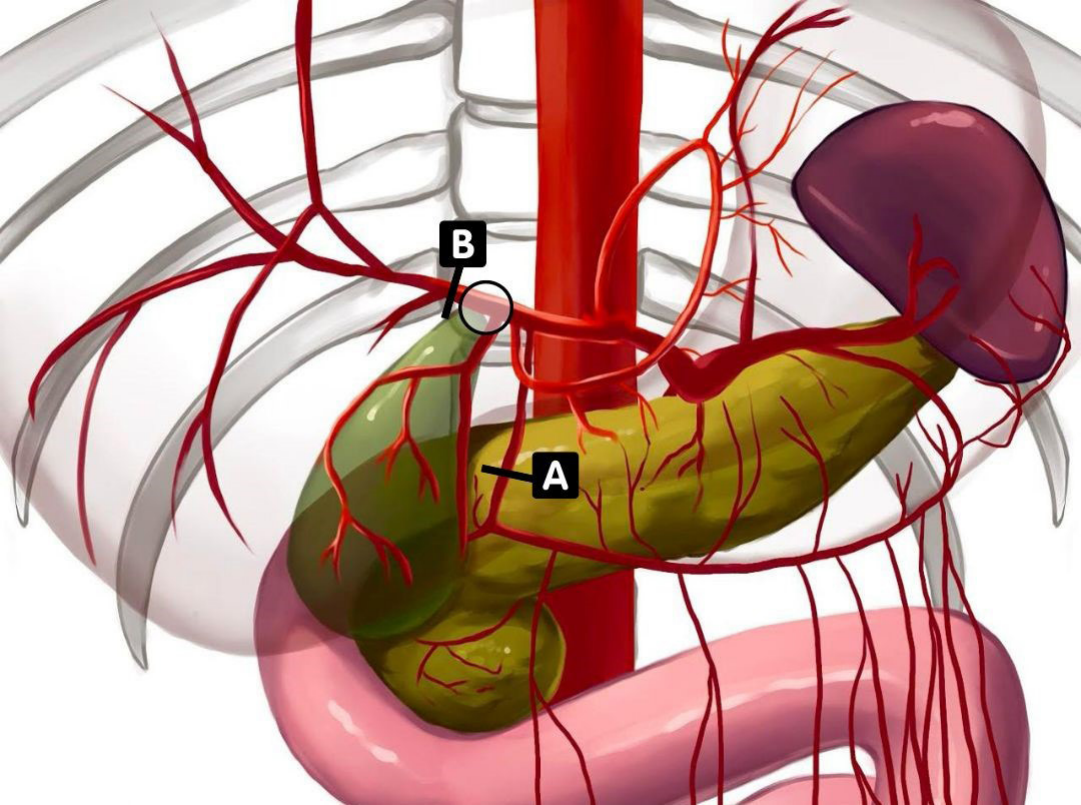
Vascular transposition of the gastroduodenal artery to the right hepatic artery. The circle illustrates the limits of the tumor with relation to the hepatic artery itself. The left hepatic artery (resected during surgery) is not shown in the image. Transposition was from (A) (gastroduodenal artery) to (B) (right hepatic artery). Author’s illustration.

**Figure 2:**
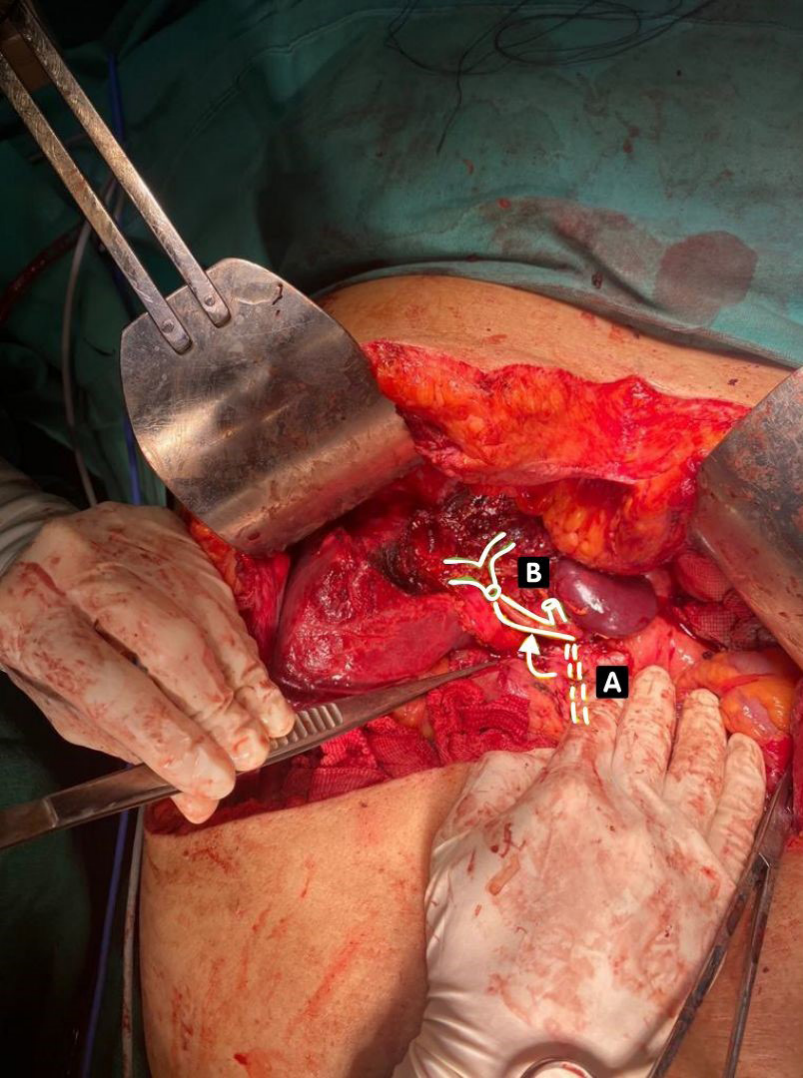
Illustration superimposed on the operating field showing the gastroduodenal artery transposed to the right hepatic artery. (A) The broken line shows the normal anatomic position; (B) The continuous line marked by the arrow shows the position after anastomosis. Authors’ files.

Surgical management was chosen and a left hepatectomy was scheduled. During surgery, an extensive hepatic mass was found in the left lobe, predominantly in hepatic segments II, III, and IV, apparently invading the gastric antrum. The tumor was in intimate contact with the gallbladder and the hepatic hilum, with total circumferential involvement of the hepatic artery proper and the bile duct. No involvement of other organs was found. The operation proceeded with dissection of the omentum, lysing of bands and adhesions, and access to the retroperitoneal cavity, delimiting the extent of gastric invasion. The hepatic mass was resected after obtaining control with a Pringle maneuver and supplemented with partial gastrectomy because of suspected invasion of the gastric antrum. The surgical specimen was removed en bloc, leaving no residual macroscopic disease. Finally, vascular transposition was conducted by anastomosis of the gastroduodenal artery to the right hepatic artery with continuous prolene 8.0 suture (Figures 1 and 2). The intestinal passage was reconstructed by bilio-digestive bypass with a transmesocolic jejunum loop, using the Roux-en-Y technique for reconstruction with end-to-side gastroanastomosis and side-to-side jejuno-jejunal anastomosis. The surgical procedure lasted 10 hours and 20 minutes and was completed without complications.

**Figure 3:**
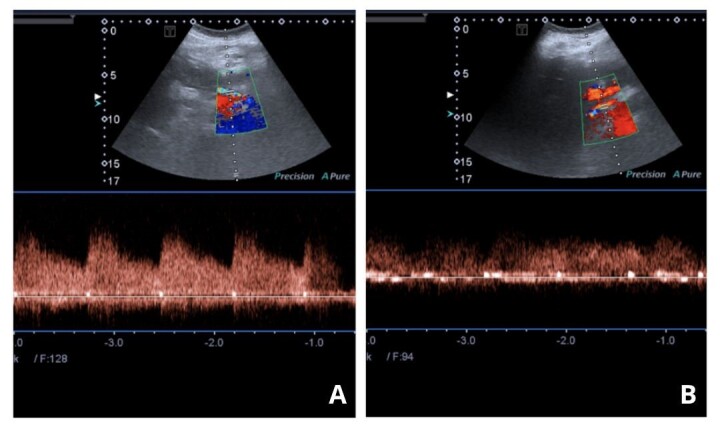
(A) Patent portal vein, with caliber maintained and Doppler velocimetry pattern preserved; (B) Surgical graft communicating between gastroduodenal and right hepatic arteries, with hepatopetal flow, no signs of turbulence, and low resistance peak systolic velocity (VPS) of 120 cm/s. Authors’ files.

Doppler ultrasound was requested and showed that the portal vein was patent with a normal Doppler velocimetry pattern (Figure 3).

The pathology report described a hepatic tumor with yellow macroscopic appearance and irregular margins, measuring 13.5 × 13.0 × 9.0 cm, 1.2 cm from the resection margin, running tangent to the capsule and causing it to bulge, and reaching a distance of 0.5 cm from the hepatic hilum. Microscopically, the tumor was defined as an adenocarcinoma with grade 2 differentiation. The gallbladder was free from neoplasm and neoplasm-free fibrous adherence to the gastric wall was observed. All 12 resected lymph nodes were free from neoplasm, as were the vascular, gastric, hepatic, and cystic duct margins.

**Figure 4:**
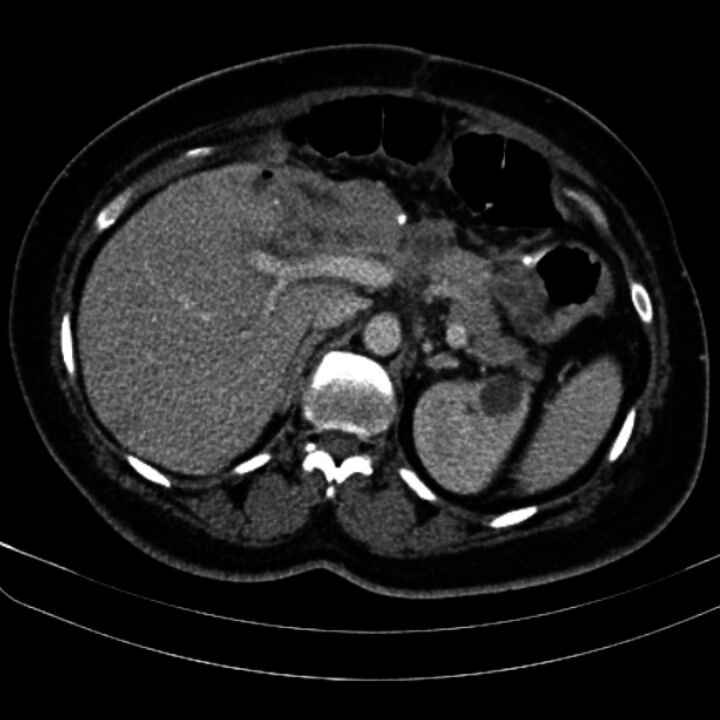
Control tomography showing status of the left hepatectomy. Authors’ files.

An abdominal tomography was requested for follow-up during the month following surgery, showing signs of partial left hepatectomy and partial gastrectomy with gastroenteroanastomosis, with the vascular graft intact (Figure 4).

Adjuvant treatment was administered with six cycles of FLOX chemotherapy (fluorouracil, leucovorin, and oxaliplatin), from February to August of 2023, without major complications. Twelve months after surgery, the patient remained in outpatient follow-up, clinically well, and with no signs of relapse on imaging exams.

## DISCUSSION

To date, vascular transposition by arterial anastomosis with autologous graft in the context of hepatectomy to treat CRC metastasis is a pioneering technique and there are no cases in the literature similar to the one described herein. A 2016 case series described 92 patients who underwent hepatectomy to treat colorectal cancer metastasis, 15 of whom underwent significant vascular resection and reconstruction. The vessels reconstructed were the portal vein, the hepatic vein, and the inferior vena cava. All reconstructions were direct and the accumulated 5-year survival rate for all patients was 54.6%, with no significant differences depending on the vascular reconstruction performed.^6^ This suggests that vascular involvement in which resection and reconstruction are feasible does not appear to negatively impact overall disease-free survival providing there are free margins.

Reconstruction with arterial anastomosis using a gastroduodenal artery graft, rather than a venous graft, was a fundamental element in the success of this case. Artery to artery anastomosis, using vessels with similar calibers and histological characteristics contributes to safety and long-term patency. This is evident in myocardial revascularization surgery, in which grafts constructed with the internal mammary artery demonstrated better long-term patency than venous grafts (saphenous vein).^7^

The anastomosis suture technique is also relevant. Of 1,050 patients who underwent liver transplantation from 2002 to 2014 at a specialist liver transplant center in Latin America, 105 were operated using the continuous vascular suture technique, with a 6.7% rate of recipient thrombosis. In contrast, 945 patients were operated using the interrupted suture technique, with a 2.5% rate of recipient thrombosis (p = 0.018).^8^ Although the vascular thrombosis rate is slightly higher for continuous sutures, the patient in the case reported here did not develop this complication. The Doppler ultrasound examination showed that the vascular anastomosis was patent and liver function was preserved 12 months after surgery. It is thought that the fact that the patient had good liver function and her liver was virgin to chemotherapy (in contrast with the hepatic dysfunction seen in patients waiting for transplantation) could have contributed to minimizing aggravating factors related to vascular thrombosis.

Use of an autologous vascular graft rather than prosthetic graft material is another important advantageous factor. Autologous grafts have greater complacency, storing energy during systole and releasing it during diastole, driving the blood flow. Prosthetic grafts have more rigid structural characteristics, reducing this secondary pulsatile force, which can reduce distal perfusion.^9^ Infection of vascular prostheses is uncommon, but it is associated with high morbidity and mortality.^10^ In general, the treatment recommended is removal of all tissue involved by infection, including the vascular prosthesis, requiring extra-anatomic revascularization.^10^ Considering the topography in which vascular transposition was performed, this would have been disastrous, with a high chance of death.

The choice of hepatectomy, whether non-anatomic or anatomic (respecting the segmentation of the liver) has not been associated with significant differences in rates of positive margin, recurrence, or survival.^11^ The smallest histopathologically neoplasm-free margin was 0.5 cm from the hepatic hilum. A study with 4,915 patients who underwent hepatectomy to treat colorectal adenocarcinoma metastasis showed that there was no statistical difference in overall long term survival when two groups were compared, the first with margins from 1 to 9 mm and the second with margins greater than 10 mm.^12^

## CONCLUSIONS

Vascular anastomosis during hepatectomy for colorectal adenocarcinoma metastasis is a delicate surgical procedure that demands skill and experience from the surgical team. The complexity of arterial anastomosis may constitute an even greater challenge and, sometimes, can limit the potential for resection of hepatic metastases. The most recent data in the literature suggest that vascular involvement that is amenable to reconstruction does not change overall disease-free survival when compared with hepatectomy without vascular reconstruction.^6^

Vascular transposition using autologous graft, respecting histological compatibility between the graft material and the recipient tissue, appears to be a factor that increases success because of its greater complacency and the lower risk of impaired distal perfusion over the long term. Further studies are needed to compare hepatectomy with or without vascular reconstruction in the context of metastasectomy for colorectal adenocarcinoma.
