# Supplementary material for: Transposition of the gastroduodenal artery for vascular reconstruction of invasive lesion of the hepatic hilum due to colorectal adenocarcinoma metastasis: case report
Source: J Vasc Bras. 2025 Jan 13;23:e20240012. doi: 10.1590/1677-5449.202400122 (PMC11758930; doi:10.1590/1677-5449.202400122)
Supplement: Translation

# Translation: Transposição da artéria gastroduodenal na reconstrução vascular de lesão invasiva do hilo hepático por metástase de adenocarcinoma colorretal: relato de caso

**DOI:** 10.1590/1677-5449.202400121

**Authors:** Lucas Lourenço de Oliveira, Iasmin Maria Rodrigues Saldanha, Yago Eloy Souza Barbosa, Renato Mazon Lima Verde Leal, Abner Moreira Sampaio, Annya Costa Araújo de Macedo Goes, Marcelo Leite Vieira Costa

**Affiliations:** 1 Universidade Federal do Ceará – UFC, Hospital Universitário Walter Cantídio – HUWC, Fortaleza, CE, Brasil.; 2 Universidade Federal do Ceará – UFC, Fortaleza, CE, Brasil.

**Keywords:** hepatectomia, neoplasias colorretais, metastasectomia, procedimentos cirúrgicos vasculares

## Abstract

O câncer colorretal é um dos cânceres mais prevalentes no Brasil. A hepatectomia para metastasectomia é essencial no aumento da sobrevida livre de doença, com a possibilidade de cura. A viabilidade da hepatectomia depende de fatores relacionados ao fígado remanescente. Neste artigo, um caso de metastasectomia hepática é reportado em uma paciente do sexo feminino, de 47 anos, portadora de adenocarcinoma colônico, submetida no mesmo ato cirúrgico à reconstrução vascular. Realizou-se a ressecção da massa intra-hepática, com a transposição da artéria gastroduodenal por meio da anastomose da artéria gastroduodenal e artéria hepática direita em sutura contínua, sem intercorrências. Conclui-se que a anastomose vascular nas metastasectomias hepáticas é um procedimento complexo que requer habilidade e experiência do cirurgião. A taxa de sucesso é elevada, mas há a possibilidade de complicações. Os dados recentes sugerem que a reconstrução vascular não altera a sobrevida global livre de doença, mas são necessários mais estudos.

## INTRODUÇÃO

O câncer colorretal (CCR) ocupa a terceira posição entre as neoplasias malignas mais prevalentes no Brasil (desconsiderando os tumores de pele não melanoma). Estima-se que venham a ser diagnosticados cerca de 45.630 novos casos entre 2023 e 2025, correspondendo a uma incidência de 21,1 casos por 100 mil habitantes^1^ . O sítio mais comum de metástase é o fígado, e dados mostram que cerca da metade dos pacientes apresentam metástases hepáticas durante sua evolução clínica^2^ .

A hepatectomia para a ressecção de metástase hepática tem um papel essencial no aumento da sobrevida livre de doença, comprovadamente com a possibilidade de cura^3^ . A sobrevivência a longo prazo após a cirurgia para metástases hepáticas do CCR melhorou significativamente. Nas últimas décadas, com o avanço das técnicas cirúrgicas, o ganho da sobrevida global em 5 anos chegou a até 64% em determinados centros^4^ .

A viabilidade da ressecção hepática depende de fatores relacionados ao fígado remanescente após a cirurgia: preservação da entrada e saída vascular com fluxo adequado; drenagem biliar; e capacidade de preservar um futuro remanescente de fígado adequado, geralmente definido como um volume de fígado remanescente de pelo menos 20% para fígado saudável e 30% após quimioterapia^5^ .

Este trabalho objetiva expor um relato de caso acerca da abordagem de uma paciente que foi submetida, no mesmo ato da hepatectomia, à transposição vascular por meio da anastomose de artéria gastroduodenal e artéria hepática direita, devido a acometimento tumoral de segmento da artéria hepática própria.

O presente artigo foi aprovado pelo Comitê de Ética em Pesquisa sob o parecer consubstanciado n° 6.619.185, com Certificado de Apresentação de Apreciação Ética: 76809324.0.0000.5045.

## RELATO DE CASO

A paciente era do sexo feminino, tinha 47 anos de idade, e buscou atendimento médico por apresentar plenitude pós-prandial, dispneia, astenia e aumento do volume abdominal há 4 meses. Tinha histórico patológico pregresso de adenocarcinoma de cólon moderadamente diferenciado (pT2N0) tratado com retossigmoidectomia em 2016, porém com perda de seguimento clínico. Retornou ao acompanhamento em 2022, quando foi realizada uma tomografia computadorizada que identificou imagem sugestiva de metástase restrita ao fígado, localizada no lobo esquerdo, medindo 15,7 cm × 10,5 cm.

Optou-se pela abordagem cirúrgica com programação de hepatectomia esquerda. No intraoperatório foi evidenciado uma extensa massa hepática no lobo esquerdo, predominantemente nos segmentos hepáticos II, III e IV, com aparente invasão de antro gástrico. A lesão apresentava íntimo contato com a vesícula biliar e o hilo hepático, com acometimento circunferencial completo da artéria hepática própria e do colédoco. Não foi evidenciado comprometimento de demais órgãos. Procedeu-se à dissecção omental, lise de bridas e aderências, e acesso à cavidade retroperitoneal com delimitação da invasão gástrica. Realizou-se a ressecção da massa hepática sob manobra de Pringle, complementando com gastrectomia parcial devido a suspeita de invasão do antro gástrico. A retirada da peça cirúrgica foi realizada em bloco sem deixar doença residual macroscópica. Por fim, a transposição vascular foi realizada por meio da anastomose da artéria gastroduodenal e artéria hepática direita com sutura contínua, utilizando fio prolene 8.0 (Figura 1 e Figura 2). O trânsito intestinal foi reconstruído mediante derivação biliodigestiva com alça de jejuno transmesocólica, utilizando a técnica de reconstrução em “Y” de Roux, com gastroanastomose término-lateral e jejuno-jejunal latero-lateral. O procedimento transcorreu durante 10 horas e 20 minutos e ocorreu sem intercorrências.

**Figura 1:** Transposição vascular da artéria gastroduodenal para artéria hepática direita. O círculo representa os limites da extensão tumoral em relação à artéria hepática própria. Artéria hepática esquerda (ressecada no ato cirúrgico) excluída da imagem. A transposição aconteceu de (A) (artéria gastroduodenal) em direção a (B) (artéria hepática direita). Imagem autoral.

**Figura 2:** Ilustração no campo cirúrgico da transposição: artéria gastroduodenal para artéria hepática direita. (A) A linha tracejada indica posição anatômica habitual; (B) A linha contínua sinalizada pela seta mostra posição após a anastomose. Acervo dos autores.

O ultrassom com Doppler foi solicitado e revelou que a veia porta estava patente, com padrão dopplervelocimétrico conservado (Figura 3).

**Figura 3:** (A) Veia porta patente, de calibre mantido e padrão dopplervelocimétrico conservado; (B) Enxerto cirúrgico comunicando as artérias gastroduodenal e hepática direita com fluxo hepatopetal, sem sinais de turbilhonamento, com velocidade de pico sistólico (VPS) de 120 cm/s de baixa resistência. Acervo dos autores.

A análise anatomopatológica revelou uma lesão hepática de aspecto macroscópico amarelado, de bordas irregulares, medindo 13,5 × 13,0 × 9,0 cm, distando 1,2 cm da margem de ressecção, tangenciando a cápsula e abaulando-a, além de distar 0,5 cm do hilo hepático. À microscopia, a lesão foi definida como adenocarcinoma com grau 2 de diferenciação. A vesícula biliar estava livre de neoplasia e foi observada presença de aderência fibrosa à parede gástrica, sem neoplasia. Os 12 linfonodos ressecados estavam livres de neoplasia, assim como as margens vascular, gástrica, hepática e de ducto cístico.

Uma tomografia de abdome de seguimento foi solicitada no mês seguinte à cirurgia e evidenciou sinais de hepatectomia parcial esquerda e gastrectomia parcial com gastroenteroanastomose, além de integridade do enxerto vascular (Figura 4).

**Figura 4:** Tomografia de controle apresentando *status* de hepatectomia esquerda. Acervo dos autores.

Foi realizado tratamento adjuvante com esquema quimioterápico de seis ciclos do FLOX (fluorouracil, leucovorin e oxaliplatina), de fevereiro até agosto de 2023, sem maiores intercorrências. Passados 12 meses desde a cirurgia, a paciente continuava em seguimento ambulatorial, clinicamente bem e sem sinais de recidiva nos exames de imagem.

## DISCUSSÃO

A transposição vascular por meio da anastomose arterial com enxerto autólogo no contexto da hepatectomia por metástase de CCR é, até o momento, pioneira, não sendo encontrado na literatura qualquer caso semelhante ao descrito. Em um estudo com uma série de casos de 2016, 92 pacientes foram submetidos à hepatectomia para tratamento de metástase de câncer colorretal, e 15 foram submetidos à ressecção e reconstrução vascular importante. Os vasos reconstruídos foram a veia porta, a veia hepática e a veia cava inferior. Todas as reconstruções foram diretas e a taxa de sobrevida acumulada em 5 anos para todos os pacientes foi de 54,6%, sem diferença significativa, de acordo com a reconstrução vascular^6^ . Esse dado sugere que o acometimento vascular passível de ressecção e reconstrução, desde que apresente margens livres, parece não interferir como agravante na sobrevida global livre de doença.

A reconstrução através da anastomose arterial utilizando enxerto da artéria gastroduodenal, em comparação ao enxerto venoso, foi um fator preponderante para o sucesso do caso. Uma anastomose artéria com artéria, utilizando-se de vasos com calibres e aspectos histológicos semelhantes, corrobora para a segurança e patência a longo prazo. Isso é evidenciado na cirurgia para revascularização miocárdica, na qual o enxerto com a artéria torácica interna demonstrou maior perviedade a longo prazo quando comparado aos enxertos venosos (veia safena)^7^ .

A técnica de sutura anastomótica também é um aspecto relevante. Entre 1.050 pacientes submetidos ao transplante hepático entre 2002 e 2014 em um centro especializado em transplantes hepáticos da América Latina, 105 foram operados com a técnica de sutura vascular contínua, apresentando uma taxa de 6,7% de trombose no receptor. Em contraste, 945 pacientes foram submetidos à técnica de sutura interrompida, com uma taxa de 2,5% de trombose no receptor (p = 0,018)^8^ . Pondera-se que, embora a taxa de trombose vascular seja ligeiramente maior em suturas contínuas, a complicação não foi apresentada pela paciente do caso aqui relatado. O exame ultrassonográfico Doppler demonstrou a patência da anastomose vascular e preservação da função hepática após 12 meses do ato cirúrgico. Considera-se que o fato da paciente apresentar fígado com boa funcionalidade e virgem de tratamento quimioterápico (diferentemente da disfunção hepática dos pacientes em perspectiva de transplante) pode ter contribuído para minimizar os fatores agravantes para o processo de trombose vascular.

A utilização do enxerto vascular autólogo em contrapartida a um enxerto protético é outro aspecto importante e vantajoso. O enxerto autólogo é mais complacente, armazenando energia durante a sístole, liberando-a durante a diástole e, posteriormente, impulsionando o fluxo sanguíneo. O enxerto protético possui uma característica estrutural mais rígida, reduzindo essa força pulsátil secundária, podendo reduzir a perfusão distal^9^ . A infecção de prótese vascular é incomum, porém está associada a uma ampla morbidade e mortalidade^10^ . De maneira geral, o tratamento preconizado é a remoção de todo o tecido acometido pela infecção, incluindo a prótese vascular, e envolve a revascularização extra-anatômica^10^ . Considerando a topografia na qual a transposição vascular foi estabelecida, isso seria desastroso e teria uma alta chance de mortalidade.

A escolha da hepatectomia entre não anatômica e anatômica (respeitando as segmentações hepáticas) não foi associada a uma diferença significativa nas taxas de margem positiva, recorrência ou sobrevivência^11^ . A margem histopatológica livre de neoplasia mais próxima distava 0,5 cm no hilo hepático. Um estudo incluindo 4.915 pacientes submetidos a hepatectomia por metástase de adenocarcinoma colorretal demonstrou que não houve diferença estatística na sobrevida global prolongada quando comparados dois grupos, sendo o primeiro com margem entre 1 e 9 mm e o segundo com margem maior que 10 mm^12^ .

## CONCLUSÃO

A anastomose vascular na hepatectomia por metástase de adenocarcinoma colorretal é um procedimento cirúrgico delicado que requer habilidade e experiência da equipe cirúrgica. A complexidade de uma anastomose arterial pode representar um desafio ainda maior e, por vezes, pode limitar a ressecabilidade das metástases hepáticas. Os dados mais atuais da literatura sugerem que o comprometimento vascular passível de reconstrução não altera a sobrevida global livre de doença quando comparado com hepatectomias sem reconstruções vasculares^6^ .

A transposição vascular com enxerto autólogo, respeitando a compatibilidade histológica do tecido utilizado e do tecido receptor, parece ser um fator potencializador do sucesso devido à maior complacência e menor risco de redução na perfusão distal a longo prazo. São necessários mais estudos de finalidade comparativa entre hepatectomias com ou sem reconstrução vascular no contexto de metastasectomias por adenocarcinoma colorretal.
